# Assessing Quality Outcome Measures in Children with Coeliac Disease—Experience from Two UK Centres

**DOI:** 10.3390/nu5114605

**Published:** 2013-11-19

**Authors:** Alexander Ross, Helen Shelley, Kim Novell, Elizabeth Ingham, Julia Callan, Robert Heuschkel, Mary-Anne Morris, Matthias Zilbauer

**Affiliations:** 1 Department of Paediatric Gastroenterology, Addenbrooke’s Hospital, Cambridge University Hospitals, Cambridge, CB2 0QQ, UK; E-Mails: Alexander.Ross@addenbrookes.nhs.uk (A.R.); Helen.Shelley@addenbrookes.nhs.uk (H.S.); Kim.Novell@addenbrookes.nhs.uk (K.N.); Julia.Callan@addenbrookes.nhs.uk (J.C.); Robert.Heuschkel@addenbrookes.nhs.uk (R.H.); 2 Department of Paediatric Gastroenterology, Norfolk and Norwich University Hospital, Norwich, NR4 7UY, UK; E-Mails: Elizabeth.Ingham@nhs.net (E.I.); Mary-Anne.Morris@nnuh.nhs.uk (M.-A.M.)

**Keywords:** quality outcome measures, coeliac disease, outpatient management, patient satisfaction

## Abstract

Improved diagnosis of coeliac disease has increased incidence and therefore burden on the health care system. There are no quality outcome measures (QOM) in use nationally to assess hospital management of this condition. This study applied QOM devised by the *East of England* paediatric gastroenterology network to 99 patients reviewed at two tertiary hospitals in the Network, to assess the quality of care provided by nurse led and doctor led care models. The average performance across all QOM was 96.2% at Addenbrooke’s Hospital (AH), and 98.7% at Norfolk and Norwich Hospital (NNUH), whilst 95% (*n* = 18) of QOM were met. Patient satisfaction was high at both sites (uptake of questionnaire 53 of 99 patients in the study). The study showed a comparably high level of care delivered by both a nurse and doctor led service. Our quality assessment tools could be applied in the future by other centres to measure standards of care.

## 1. Introduction

Coeliac disease affects up to 0.5%–1% of all children in Europe and North America, with a large number of cases being undiagnosed [1,2,3]. With the implementation of simple widespread diagnostic screening tools, the incidence has increased rapidly in recent years, leading to an increasing burden on the healthcare system [3].

Overall, it is very well established that a lifelong gluten free diet results in symptom resolution and normalisation of tissue transglutaminase (tTG) in the majority of patients [4,5], and remains the mainstay of treatment. Hence, patient care includes the involvement of primary, secondary and tertiary healthcare institutions as well as several health care professionals such as dieticians, nurses, and doctors. The ultimate aim is to optimise resources and expertise in order to provide the highest quality patient care.

In the East of England, patients are managed within the *East of England Paediatric Gastroenterology Network (EEPGN)*, which operates two tertiary centres (Addenbrooke’s hospital, Cambridge University Hospitals, and Norfolk and Norwich University Hospital) and fifteen secondary care sites. Approximately 100 patients with coeliac disease are reviewed annually at Addenbrooke’s hospital (AH) and 100 at Norfolk and Norwich University Hospital (NNUH) in specialist “coeliac clinics” run on a monthly basis at each site. These clinics are set up to deliver a review of coeliac related symptoms and general health, assessment of growth and nutritional status, understanding, adherence and support to optimise compliance with the diet. Food diaries are completed prior to each clinic, with blood results taken prior to each clinic so that results are available during the consultation. Following diagnosis, patients are reviewed as outpatients at three, six, and twelve months, and annually thereafter. At NNUH annual review is conducted by Doctors and Dieticians, however at AH review is predominantly run by Dieticians and Specialist Nurses, with doctors available for consultation if required. Once patients reach the age of sixteen years, their care is transitioned on to the adult gastroenterology or primary care services.

Assessment of Quality Outcome Measures (QOM) is growingly important to assess efficiency and quality of service delivery, however there are currently no formally stated national QOM for paediatric coeliac disease. It has previously been shown that measures of care quality can be beneficial in both acute [6], and chronic [7] conditions in Paediatric care, and that quality of care correlates with patient satisfaction [8]. The main aim of our study was to use a set of QOM devised by the EEPGN, which were based upon national guidelines [5] to assess quality of care delivered by each hospital. The QOM were developed and approved by the EEPGN steering group comprising Paediatricians, Paediatric Gastroenterologists, Paediatric Specialist Nurses and Dietitians with representation from the two tertiary and five of the secondary care units. The QOM were designed to represent acceptable quality of care, be patient centered and be applicable to a range of service models across tertiary and secondary units. These QOM were then used to assess the quality of care and patient/parent satisfaction at the two main tertiary centres each offering a different service care model.

## 2. Experimental Section

All patients (*n* = 99) seen in the annual review clinic at both centres during a six month period (01/11/10 to 31/05/11) were included in the study. Three major areas of QOM were assessed; diagnosis and initial patient management, the annual clinical review process, and transition of care from paediatrics to adult medicine.

The “annual clinical review process” and “transition of care from paediatrics to adult medicine” data were collected using a proforma completed during the clinic session. The proforma was designed to reflect the 2009 NICE guidelines (CG86) for the management of coeliac disease [5]. The proforma consisted of thirteen questions regarding patient care, and was completed by the assessing clinicians during each appointment (Appendix 1). The Data relating to each patient’s “diagnosis and initial management” was extracted retrospectively from patient documentation using an extraction tool based consisting of the six areas outlined for this subsection (Appendix 1).

“Patient satisfaction” was assessed using an anonymised questionnaire designed by paediatric gastroenterologists, dieticians, and nurse specialists within the East of England Paediatric Gastroenterology network, which was completed by parents of children attending an annual review clinic. Parents were allowed to complete each questionnaire in their own time after the clinic appointment had taken place, and handed in the data upon completion. There were eleven questions included in the questionnaire. Uptake of the questionnaire was 53 of the 99 (54%) patients included in the study (AH *n* = 20, NNUH *n* = 33). Each assigned QOM had a predefined target of 90% concordance.

A table of each area assessed, and the outcome for each area can be found in Appendix 1.

The original data was collected as part of a clinical audit and therefore in accordance with Department of Health (UK), did not require formal ethical approval [9].

## 3. Results

### 3.1. Quality Outcome Measures

The initial focus of our investigation was to compare the quality outcome measures at each site to assess the quality of care delivered by each system, i.e., nurse and dietician (AH) versus doctor and dietician (NNUH) led care. The average performance across all QOMs was 96.2% at AH, and 98.7% at NNUH.

**Figure 1:**
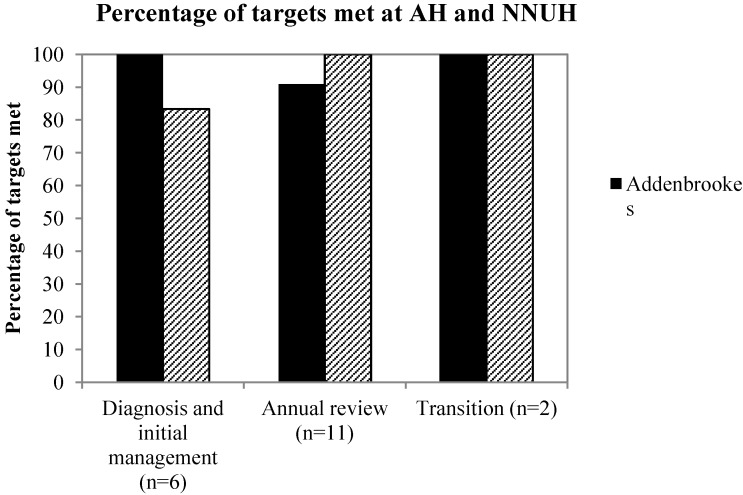
Comparison of the percentage of targets reached in each quality outcome measures (QOM) section between Addenbrooke’s Hospital (AH) (solid bars), and Norfolk and Norwich Hospital (NNUH) (hashed bars), showing the majority to targets being reached in each section.

For “diagnosis and initial management”, six areas were assessed, with the average achievement in each standard as 96.3% at NNUH, and 92.3% at AH. As demonstrated in Figure 1, AH met the 90% target set in each subsection, whilst NNUH achieved compliance in five of the six targets (Figure 1). The QOM target that was not achieved was “performance of biopsy within four weeks of positive serology result”.

To assess the quality delivered at the “annual clinical review of patients” eleven areas were assessed. The average percentage of compliance across each standard was 98.3% at AH, and 100% at NNUH. NNUH therefore met the 90% target in each of the eleven QOMs, however AH did not achieve the target, “tTG less than twice upper limit of normal within two years of diagnosis” (Figure 1).

Efficient referral of children to adult services is essential as it preserves continuity of ongoing care and provides vital information to the adult team. To assess the quality of patient referral at AH and NNUH two QOMs were assessed, “discussion of referral”, and “completed referral before sixteen years of age”. There were two referral cases at AH, and five at NNUH. In 100% of cases at AH and NNUH, referral was discussed during a clinic appointment. Of the five cases at NNUH, four were appropriately referred to adult services, however in one exceptional case at NNUH it was decided with the patient’s carer that the child should remain under paediatric care for the next year due to multiple significant co-morbidities.

### 3.2. Parent and Patient Satisfaction

**Figure 2:**
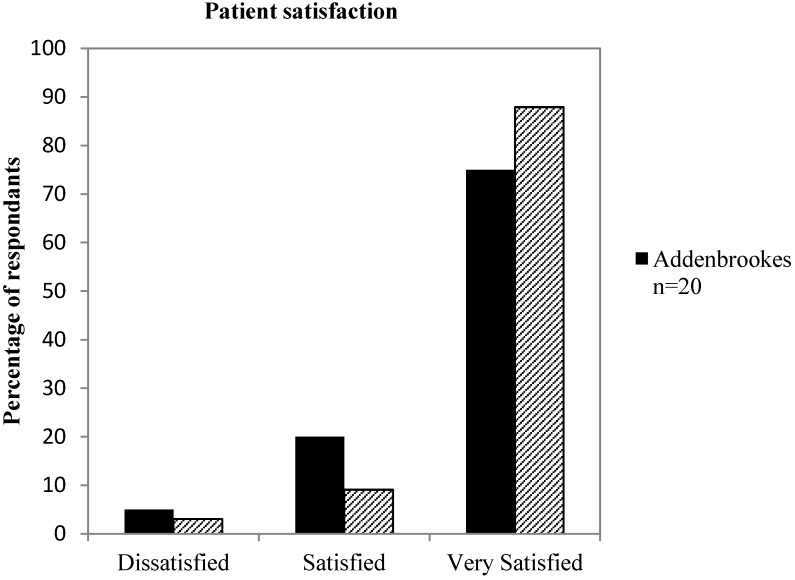
Comparison of parent satisfaction levels at AH (solid bars) and NNUH (hashed bars). Satisfaction levels are mostly very high in both sites, with slightly higher levels noted in NNUH.

In addition to the EEPGN quality of care assessment, it is important that parents and patients perceive the healthcare service provided as being of a high standard. Therefore, a parent and patient satisfaction survey was used to assess the satisfaction at each site. The overall satisfaction ratings were comparably high at both sites, with 88% (*n* = 29) of responses at NNUH and 75% (*n* = 15) at AH rated as “very satisfied” (Figure 2). 3% (*n* = 1) of responses at NNUH and 5% (*n* = 1) at AH were rated as dissatisfied, and in all cases the cause listed was long waiting times when attending clinic.

**Figure 3:**
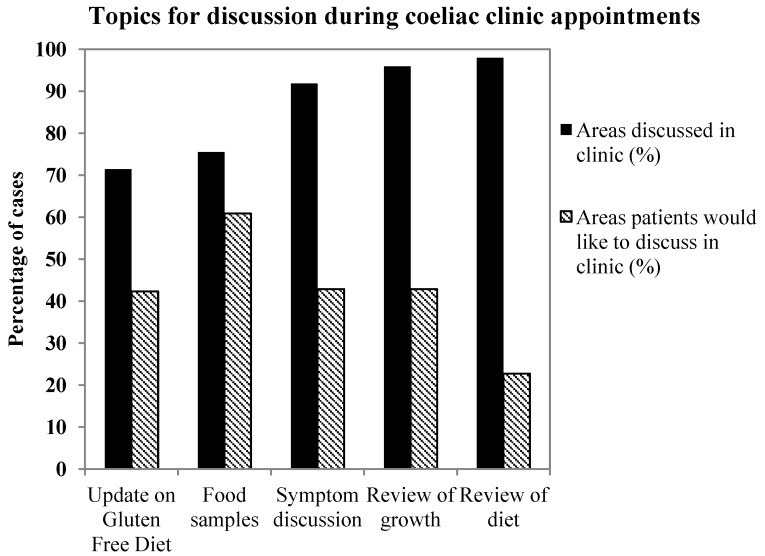
Bar graph displaying the percentage of topics patients would like to discuss (hashed bars) contrasted to the areas actually discussed during the appointment. Data contains answers from all patients both at AH and NNUH.

The combined data for AH and NNUH showed that the best reviewed areas during consultation were patient diet (98%, *n* = 52), symptoms (92%, *n* = 49), and growth (96%, *n* = 51) (Figure 3). The less well covered areas were updating patients about gluten free diet options, and availability of food samples. Interestingly, when questioned on which areas parents would like to be covered, the greatest response was for gluten free food samples to be provided during the appointment (61%). The area that parents least wanted to discuss was review of diet (23%).

## 4. Discussion

Numbers of children and adolescents diagnosed with coeliac disease are rapidly increasing in most European countries including the UK [3]. Given substantial financial restrictions to most healthcare systems new cost-effective solutions to provide high quality patient care are urgently required. Addenbrooke’s hospital adopted an alternative approach to the doctor led coeliac clinic by running a predominantly dietician and nurse led service with doctors offering advice in specific circumstances. In contrast, a second tertiary centre in the east of England ran the model of a primarily doctor led service. In this study we assessed and compared the quality of both models using QOM and parent satisfaction. Results of our study demonstrate that both approaches provide similarly high standards of care.

The missed target in the “diagnosis and initial management” subsection was “diagnostic biopsy within four weeks of positive serology at NNUH”. This was due to waiting times for endoscopy, preventing more prompt scheduling of biopsy. Our data has prompted a review of patient pathway to meet this target. Subsequent to our study there have been changes to the diagnostic guidelines provided by the British Society of Paediatric Gastroenterology, Hepatology and Nutrition (BSPGHAN) allowing the diagnoses of coeliac disease to be made without the requirement of a small bowel biopsy. This has the potential to improve endoscopic capacity in both units as well as delivering cost savings to the healthcare economy [10].

Within the “annual clinical review of patients” subset of QOM, the only missed target at the two sites was to achieve a tTG value less than twice the upper limit of normal within two years. This was the case for five patients at AH, despite NNUH achieving 100% in this target. The most likely cause of elevated tTG following diagnosis is poor adherence to a gluten free diet as refractory coeliac disease is rare [4]. As a learning point arising from our data, increasing focus has now been placed on the provision and support for parents and patients in adhering to a gluten free diet at AH further highlighting the vital role of nurse specialists and dieticians in the long term management of children with coeliac disease.

Uptake of the voluntary questionnaire was low across both centres, and was lower at AH (41%, *n* = 20) than NNUH (66%, *n* = 33). It is possible that patients and their parents that had received a low quality of service might not have wanted to give feedback, and this must be considered when comparing the highly positive feedback at the two centres. Excellent overall satisfaction ratings were recorded at both sites, and this correlation with good QOM scores reflects previous research into patient satisfaction [8]. The cause stated for all dissatisfied parents was waiting times, which were up to two hours long. Waiting times can be very difficult to control in the clinical setting, however given that 50% of patients would be happy to be seen in a non-coeliac clinic, it may be possible to further outsource patient care according to specific requirements such as dietetic advice or practical guidance, which could be provided by trained general practitioners, healthcare visitor or nurse specialists. Care could then be reassessed using further application of QOM.

An additional area that was found to carry potential for future service improvement was the topic of clinic consultation. Specifically, parents most frequently voiced the desire for gluten free food samples to be provided during clinic. In contrast, the need to discuss and review current dietary issues was much less pertinent to parents in the study, whilst the dietician’s impression of nutritional adequacy and gluten exclusion implied this was an important issue. Providing a large variety of gluten free food options is essential in aiding the ability of patients to adhere to a gluten free diet as well as improving overall quality of life. However the financial burden of Coeliac Disease to families and health care economy is considerable [11], and in our experience children’s taste preferences for products are hard to predict. The provision of samples of new and different products for children to try before requesting on prescription could potentially improve compliance and reduce wastage. Hence, it may be beneficial for the future development of the service to explore the options of working together with industry in providing a greater exposure to new products on the market.

What defines excellence in care provision is still open to discussion, and the relative importance in each QOM in reflecting care quality remains subjective. The conclusions of this study are also limited by the study size; future investigation into care quality would benefit from studying patient care over a longer period of time, allowing for more detailed analysis of the cohort and estimation of cost saving. Additionally, guidelines are subject to change and therefore QOM would need to be regularly assessed and redesigned to reflect such changes.

## 5. Conclusions

In summary, using predefined QOM we were able to demonstrate that an equally high standard of care for children with coeliac disease can be delivered both via a nurse and dietician led, and a doctor and dietician led service. The QOM and parent satisfaction results also offer further insight into ways of further developing the service to meet the demands of an increasing patient population. We have now extended our study to several secondary care sites within the EEPGN, with preliminary data suggesting comparably high outcomes at these sites.

## Appendix

**Appendix 1:** Summary table of each quality outcome measure and percentage of cases in which each measure was met at AH, NNUH, and the target set for both sites.

		Percentage cases in which standards met		
	
		AH	NNUH	Target
**1**	**Diagnosis and initial management**			
i	Biopsy within 4 weeks of referral	90	78	90
ii	Carer informed within 5 days	100	100	90
iii	Dietetic review within 2 weeks	91	100	90
iv	Information pack sent within 2 weeks	91	100	90
v	Coeliac UK	91	100	90
vi	Team details provided	91	100	90
**2**	**Annual review**			
i	Annual appointments offered	100	100	90
ii	Annual attendance	94	100	90
iii	Non-attenders seen within 6 months	100	100	90
iv	Proforma completed	100	100	90
v	Understanding of GFD assessed	98	100	90
vi	GF prescriptions reviewed	100	100	90
vii	Growth assessed	100	100	90
viii	Bloods taken annually	100	100	90
ix	tTG less than twice upper limit of normal within 2 years	89	100	90
x	Coeliac UK membership	100	100	90
xi	Iron and calcium intake	100	100	90
**3**	**Transfer of care**			
i	Transition discussed	100	100	90
ii	Smooth transition process	100	100	90
